# Maternal and neonatal outcomes of twin pregnancies complicated by gestational diabetes mellitus

**DOI:** 10.1007/s12020-023-03588-0

**Published:** 2023-11-10

**Authors:** Zhengyu Zhang, Lingwei Mei, Li Li, Jumei Xiao, Xiaoxin Wu, Yuan Yuan

**Affiliations:** 1 Medical Records Department, The First Affiliated Hospital, Zhejiang University School of Medicine, Hangzhou, 310003 China; 2 Department of Obstetrics and Gynecology, Women and Children’s Hospital of Chongqing Medical University, Chongqing Health Center for Women and Children, Chongqing, 401147 China; 3 State Key Laboratory for Diagnosis and Treatment of Infectious Diseases, National Clinical Research Centre for Infectious Diseases, The First Affiliated Hospital, Zhejiang University School of Medicine, 79 Qing Chun Road, Hangzhou, 310003 Zhejiang China; 4 Medical Department, Women and Children’s Hospital of Chongqing Medical University, Chongqing Health Center for Women and Children, Chongqing, 401147 China

**Keywords:** GDM, Twin pregnancy, Preterm birth, Insulin

## Abstract

**Introduction:**

Gestational diabetes mellitus (GDM) is associated with a higher risk of adverse maternal outcomes, but its effects on maternal and perinatal outcomes of twin pregnancies remain conflicting.

**Methods:**

This retrospective cohort study included all primipara who delivered twin pregnancies at a single tertiary perinatal center between January 1, 2016 and December 31, 2022. Excluded were those who had a single pregnancy, twin pregnancies with pre-existing diabetes, missing information on GDM screening, a delivery before gestational 28 weeks, complications related to monochorionic placentation, multifetal reduction, fetal anomalies, and monochorionic monoamniotic twins. Maternal outcomes included preterm birth, pre-eclampsia, hypothyroidism, preterm premature rupture of membranes (PROM), placental abruption, severe postpartum hemorrhage, and oligohydramnios. Neonatal outcomes included small-for-gestational-age (SGA), large-for-gestational-age (LGA), birthweight, Apgar score, neonatal intensive care unit (NICU) admission, extrauterine growth restriction (EUGR), and neonatal hypoglycemia.

**Results:**

A total of 3269 twins were delivered, with 897 women (27.4%) diagnosed with GDM during pregnancies; moreover, 72 (8.0%) of these women received insulin treatment. The GDM group showed a significantly higher maternal age at delivery (≥35 years), as well as incidences of overweight and obesity. These factors also elevated the odds of insulin treatment in GDM women with twin pregnancies (OR = 1.881, 95% CI = 1.073–3.295, *P* = 0.027; OR = 2.450, 95% CI = 1.422–4.223, *P* < 0.001; OR = 4.056, 95% CI = 1.728–9.522, *P* < 0.001, respectively). Chronic hypertension prior to pregnancy was identified as a risk factor for GDM during twin pregnancies (OR = 1.896, 95% CI = 1.290–2.785, *P* < 0.001), although it did not increase the proportion of women requiring insulin treatment (*P* = 0.808). Aside from a higher incidence of preterm birth before 37 weeks in insulin-treated GDM twins (OR = 2.096, 95% CI = 1.017–4.321, *P* = 0.045), there were no significant difference in other maternal outcomes (preterm birth before 34 weeks, pre-eclampsia, hypothyroidism, PROM, placental abruption, placenta previa, severe postpartum hemorrhage, and oligohydramnios) between the GDM group and non-GDM group, and between insulin-treated GDM and non-insulin-treated GDM. The rate of newborns with birthweight <1500 g was significantly lower among twins born to GDM women, but the prevalence of EUGR was notably higher. Additionally, the risk of EUGR was elevated in insulin-treated GDM twins (OR = 3.170, 95% CI = 1.639,6.131, *P* < 0.001). No significant differences were observed between the GDM group and non-GDM group, or between insulin-treated GDM and non-insulin-treated GDM group in terms of mean birthweight, newborn sex ratio, and incidences of other adverse neonatal outcomes, including gestational age at delivery, LGA, birth weight <2500 g, and 1-min and 5-min Apgar scores.

**Conclusion:**

Maternal age ≥35 years, overweight or obesity, and chronic hypertension are significant risk factors for GDM during twin pregnancies. Women with GDM during twin pregnancies may achieve similar outcomes compared to those without GDM. However, the women with GDM during twin pregnancies receiving insulin therapy may have a higher risk of preterm birth and EUGR.

## Introduction

Gestational diabetes mellitus (GDM), characterized by blood glucose dysmetabolism during pregnancy, involves 6–25% of pregnant women, the incidences of which vary with diagnostic criteria [1]. GDM increases not only maternal complications, such as pre-eclampsia, premature rupture of membranes (PROM), placental abruption, and postpartum hemorrhage, but also adverse perinatal outcomes, including macrosomia, intrauterine growth retardation (IUGR), neonatal hypoglycemia, and neonatal respiratory distress syndrome [2, 3]. Mounting evidence suggests that high body mass index (BMI), advanced maternal age, and assisted reproductive technology (ART) are risk factors for the occurrence of GDM [1, 4–6].

The incidence of twin pregnancies has increased with the popularity of ART worldwide [7]. Compared with singleton pregnancies, twin pregnancies increase the risks of both GDM and adverse maternal outcomes [8, 9]. However, evidence is conflicting about the effects of GDM during twin pregnancies on maternal and perinatal outcomes. Some studies have shown that GDM increases the risk of preterm birth [8–12], and GDM during twin pregnancies is associated with a higher incidence of adverse maternal outcomes, such as gestational hypertension [12, 13], pre-eclampsia [11, 13–15], cesarean deliveries [4, 8–11], as well as adverse neonatal outcomes, such as LGA [11, 13], hypoglycemia [9, 10], hyperbilirubinemia [8, 9], respiratory morbidity [8, 9], and admission to the neonatal intensive care unit (NICU) [5, 10, 11, 16].

However, other studies have found no significant associations, or even proposed that fewer adverse outcomes may result from GDM during twin pregnancies [6, 15–17]. A retrospective cohort study [6] has found that GDM during twin pregnancies does not increase the risks of preterm birth before 37 weeks, LGA, and neonatal respiratory distress (NRDS). Dave et al. [15] have found no association between the GDM during twin pregnancies and the incidence of LGA or NICU admissions. A meta-analysis by McGrath et al. [16] has indicated that GDM during twin pregnancies does not increase the probability of adverse outcomes, other than admission to NICU in newborns, after excluding the effects of BMI and maternal age. In a study by Okby et al. [17], after adjustment for confounding factors, such as maternal age, fertility treatments and hypertensive disorders, GDM during twin pregnancies shows no associations with adverse perinatal outcomes, but increases the odds of cesarean delivery. Even in twin newborns born to GDM women, both 5-min Apgar score and perinatal mortality. In addition, there may be an interaction between twin pregnancy and hyperglycemia. Previous studies mainly focused on populations in the United States and Europe, rarely China.

Herein, we conducted the following retrospective study to explore the impact of GDM during twin pregnancies on the maternal and perinatal outcomes in Chinese.

## Materials and methods

### Study design and participants

**Fig. 1:**
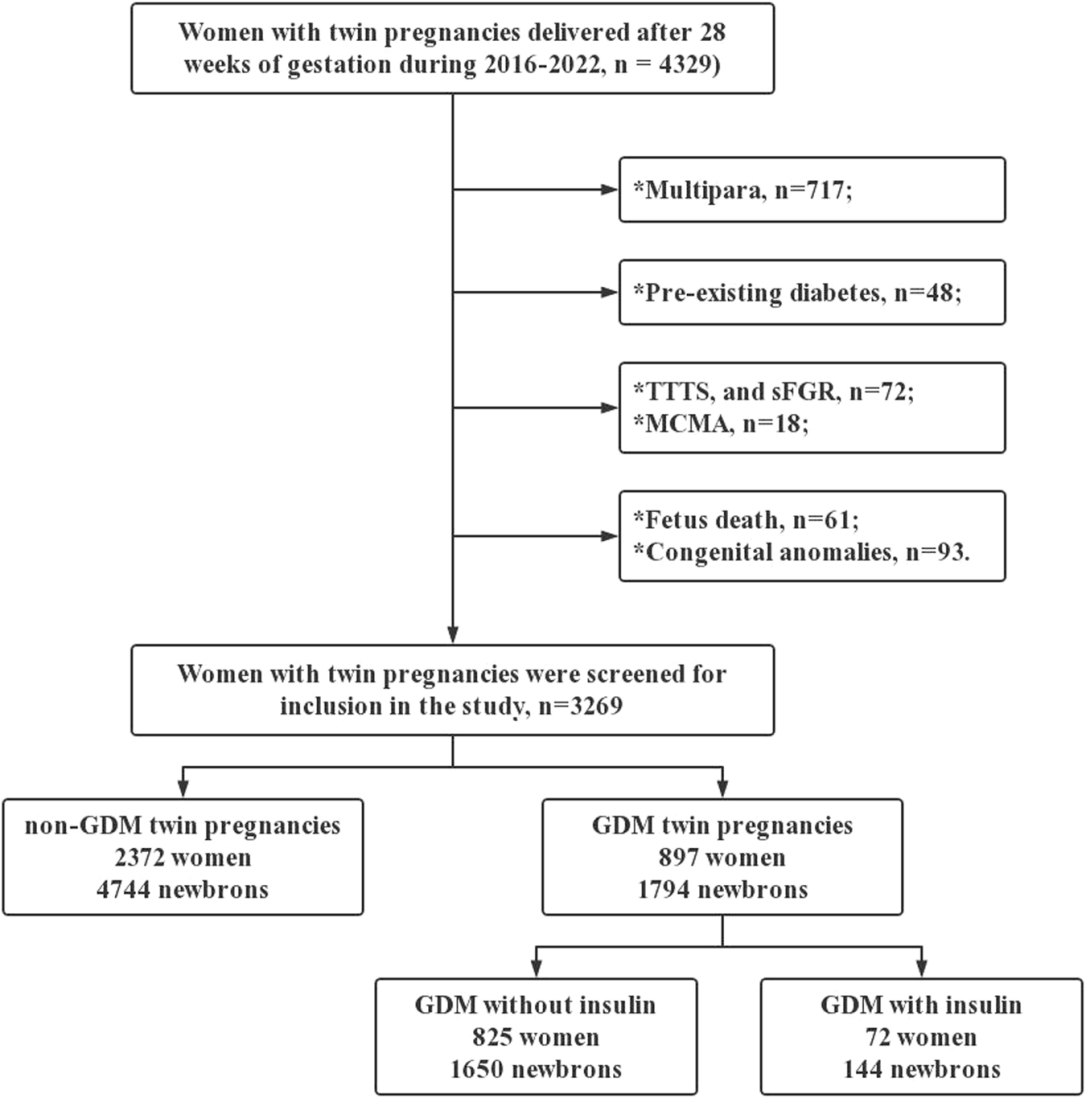
Flow chart of selection of the study population

Included into this retrospective cohort study were all the women who carried twin pregnancies and gave birth at a large tertiary perinatal center in southwestern China between January 1, 2016 and December 31, 2022. The exclusion criteria included: (1) single pregnancy; (2) lack of first-trimester ultrasound examination for chorionicity or gestational age determination; (3) pre-existing diabetes; (4) missing GDM screening results; (5) birth before gestational 28 weeks; (6) complications related to monochorionic placentation, such as twin-to-twin transfusion syndrome and selective fetal growth restriction; (7) monochorionic monoamniotic twins; (8) reduction in fetuses; and (9) fetal chromosomal or structural anomalies. To avoid confounding effects from pregnancy, only the first delivery during the study period was analyzed. Figure 1 illustrates the selection process of study participants. The institutional review board of Chongqing Health Center for Women and Children approved this study.

### Data source

The study data were obtained from institutional medical record databases, containing maternal demographics, medical and obstetric history, current pregnancy information (including complications), and perinatal outcomes. For spontaneously conceived twins, gestational age was determined by estimating the last menstrual period, and confirmed or corrected by measuring fetal crown-rump length using ultrasound between 11 and 13 + 6 weeks of pregnancy. Gestational age of twins conceived by ART was calculated solely on the date of embryo implantation. The chorionicity of twins was determined by ultrasound before 14 weeks. Dichorionic twins were diagnosed if a positive twin peak sign was present at the junction of the fetal membrane and placenta, or if two separate placentas were observed, according to the criteria presented in the literature [18].

### GDM diagnosis and glycemic control

All pregnant women, except those with pre-existing diabetes, were screened for GDM between 24 and 28 weeks of gestation, using a 75-gram oral glucose tolerance test (OGTT). An abnormal result was defined as any of the following: fasting or pre-meal glucose level ≥5.1 mmol/L or 92 mg/dL, 1-h post-meal glucose level ≥10.0 mmol/L or 180 mg/dL, or 2-h post-meal glucose level ≥8.5 mmol/L or 153 mg/dL. Women diagnosed with GDM at the center received self-monitoring blood glucose (SMBG) measurements and individualized diet-and-exercise management. The team caring for these women included maternal-fetal medical experts, nutritional consultants, and diabetes specialist nurses who provided individualized follow-up care during the entire pregnancy. Pregnant women with GDM monitored their blood glucose levels four times daily, and received insulin therapy if their levels did not meet the guidelines after diet-and exercise-management. The SMBG targets were set at fasting level ≤5.3 mmol/L or 95 mg/dL and post-meal level ≤6.7 mmol/L or 121 mg/dL, respectively.

### Maternal and neonatal outcomes

The following adverse maternal and neonatal outcomes were reported in our study:

1. Pre-pregnancy BMI [19]: Underweight, BMI < 18.5; normal weight, 18.5 ≤ BMI < 24; overweight, 24 ≤ BMI < 28; obesity, BMI ≥ 28.
2. Excessive gestational weight gain [20, 21]: Total weight gain (TWG) or gestational gain BMI (GWGR) exceeded the established standards: underweight and normal weight, TWG ≥ 21 kg or GWGR ≥ 0.58; overweight, TWG ≥ 20 kg or GWGR ≥ 0.55; obesity, TWG ≥ 18 kg or GWGR ≥ 0.50.
3. Preterm birth: before 34 or 37 complete weeks of gestation.
4. Pre-eclampsia: (I) gestational hypertension: two recordings of systolic blood pressure ≥140 mmHg or diastolic blood pressure ≥90 mmHg at least 4 h apart after 20 weeks of gestation; (II) proteinuria: excretion of urinary protein ≥300 mg in 24 h, or random urine protein at least 1+.
5. Hypothyroidism: pregestational hypothyroidism; serum thyrotropin (TSH) during pregnancy was higher than the upper limit of the reference value (4 mIU/L), and free thyroxine (FT_4_) was decreased.
6. PROM: rupture of membranes before onset of labor.
7. Placental abruption: premature separation of a normally implanted placenta before delivery of fetus.
8. Severe postpartum hemorrhage: blood loss ≥1000 mL.
9. Oligohydramnios: amniotic fluid volume ≤2 cm.
10. Small-for-gestational age (SGA) or large-for-gestational age (LGA) infants: neonatal birth weight corrected for fetal sex and gestational age below the 10th percentile of the standard value or above the 90th percentile.
11. Birthweight <1500 g or 2500 g.
12. 1-min and 5-min Apgar score <7.
13. NICU admission: admission to the NICU.
14. Extrauterine growth restriction (EUGR) was determined when, despite early and aggressive nutritional interventions, the development indicators of premature neonates remained below the 10th percentile of the expected intrauterine development levels.
15. Neonatal hypoglycemia: hypoglycemia with a plasma glucose level of <40 mg/DL.

### Statistical analysis

Maternal and perinatal data were subjected to a descriptive analysis. Categorical variables were presented as counts and percentages (*N*%), while continuous variables were expressed as means with standard deviations (mean ± standard deviation). To compare pregnancy outcomes, a Mann-Whitney test was employed for quantitative variables, and a chi-squared test or a Fisher’s exact test for categorical variables. To identify independent risk factors associated with GDM in women during twin pregnancies, we conducted a multiple logistic regression analysis. The potential risk factors considered in this analysis included maternal age, pre-pregnancy BMI, chorionicity, conception through assisted reproductive technology, and the presence of certain medical conditions (chronic hypertension, hypothyroidism, and pre-eclampsia). All analyses were run on IBM SPSS Statistics software (version 25.0). Two-tailed p-values less than 0.05 were statistically significant.

## Results

### Characteristics of study population

**Fig. 2:**
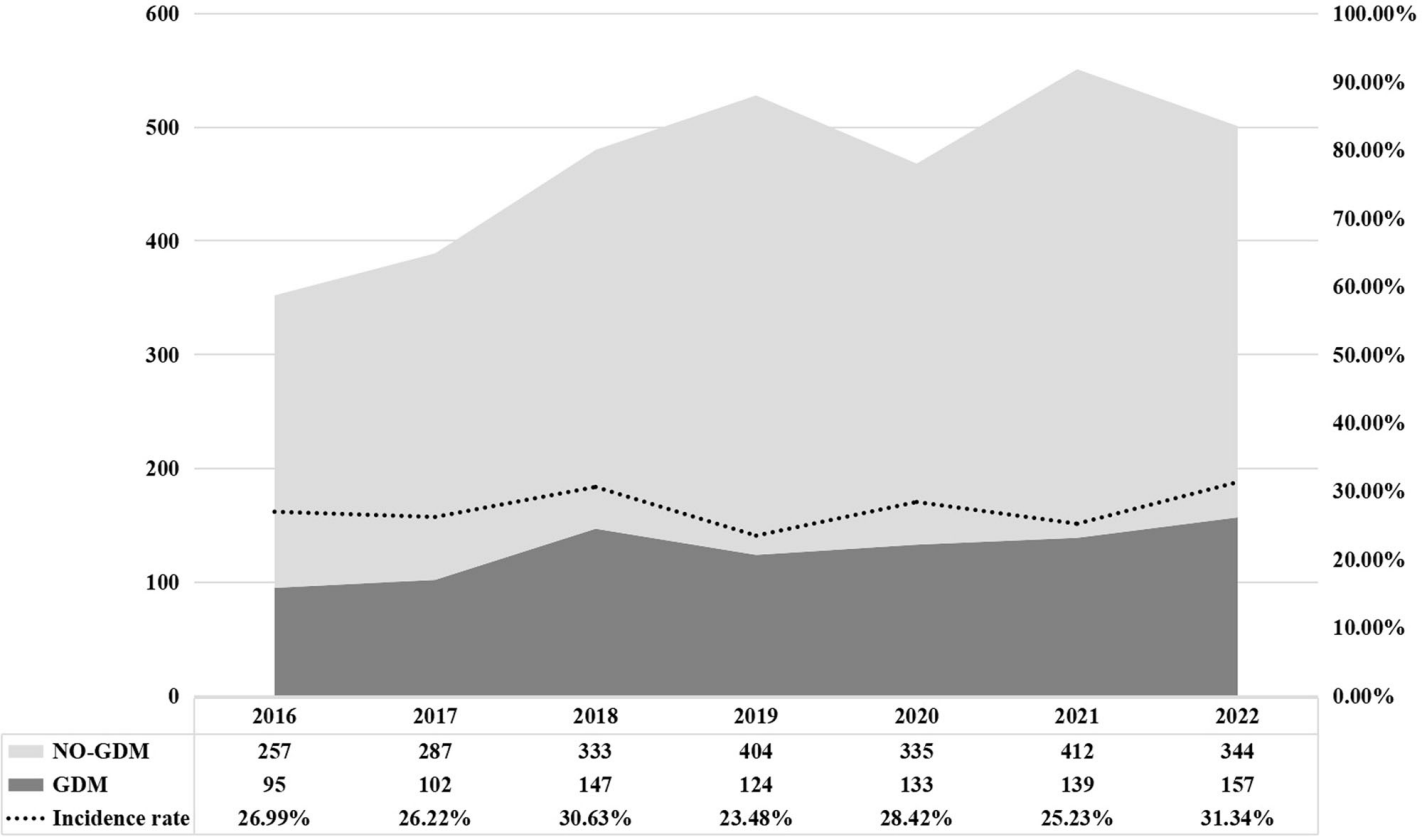
The annual incidence of GDM in twin pregnancies

The study included 3269 twins, of which 2809 (85.9%) were dichorionic diamniotic (DCDA) and 460 (14.1%) were monochorionic diamniotic (MCDA). In total, 897 pregnant women (27.4%) were diagnosed with GDM, and all received diet-and-exercise management, nutritional therapy, and regular blood glucose monitoring. Of these, 72 women (8.0%) required insulin therapy. Figure 2 shows the annual incidence of GDM during twin pregnancies during the study period.

Table 1A presents the maternal and neonatal outcomes of the study population. The rates of maternal age at delivery ≥35 years (17.9% VS 13.2%, *P* < 0.001), overweight (21.9% VS 13.7%, *P* < 0.001) and obesity (4.4% VS 1.4%, *P* < 0.001) were notably higher in GDM twin pregnancies than in non-GDM twin pregnancies. Additionally, the women with GDM during twin pregnancies exhibited significantly higher pre-pregnancy BMI values (22.39 ± 3.09 VS 21.21 ± 2.67, *P* < 0.001), and lower gestational gain BMI levels (6.44 ± 2.40 VS 6.89 ± 2.12, *P* < 0.001). Our further analysis of gestational weight gain in different pre-pregnancy BMI groups revealed that women with GDM experienced lower gestational weight gain, in comparison to women without GDM during twin pregnancies (Supplementary Table 1). There was no significant difference in GDM incidence between DCDA twins and MCDA twins; however, the incidence of GDM was significantly higher in women achieving twin pregnancies using reproductive technology (82.5% VS 78.0%, *P* = 0.005). Women with chronic hypertension before pregnancy were more likely to develop GDM (6.0% VS 2.7%, *P* < 0.001). Furthermore, we did not observe significant differences in other maternal outcomes between women with and without GDM during twin pregnancies, including preterm birth before 34 or 37 weeks of gestation, pre-eclampsia, hypothyroidism, PROM, placental abruption, placenta previa, severe postpartum hemorrhage, and oligohydramnios (Table 1B).

**Table 1:** (A) Maternal characteristics of the study population (*n* = 3269). (B) Pregnancy outcomes of the study population (*n* = 3269). (C) Maternal characteristics of women with GDM twin pregnancies (*n* = 897). (D) Pregnancy outcomes of the study population (*n* = 897)

(A)			
Maternal characteristics	Non-GDM (*n* = 2372)	GDM (*n* = 897)	*P* value
Age (SD), year	30.31 ± 3.81	31.40 ± 3.62	<0.001
< 35, *N* (%)	2061 (86.9)	735 (81.9)	<0.001
≥ 35, *N* (%)	311 (13.2)	162 (17.9)	
Chorionicity			
MCDA, *N* (%)	343 (14.5)	116 (12.9)	0.284
DCDA, *N* (%)	2029 (85.5)	781 (87.1)	
Pre-pregnancy (SD), (kg/m^2^)	21.21 ± 2.67	22.39 ± 3.09	<0.001
Underweight, *N* (%)	352 (14.8)	68 (7.6)	<0.001
Normal weight, *N* (%)	1662 (70.1)	593 (66.1)	0.029
Overweight, *N* (%)	325 (13.7)	196 (21.9)	<0.001
Obesity, *N* (%)	33 (1.4)	40 (4.4)	<0.001
Gestational gain (SD), (kg/m^2^)	6.89 ± 2.12	6.44 ± 2.40	<0.001
Excessive gestational weight gain, *N* (%)	655 (27.6)	166 (18.5)	<0.001
Conception by ART, *N* (%)	1850 (78.0)	740 (82.5)	0.005
Chronic hypertension, *N* (%)	64 (2.7)	54 (6.0)	<0.001

(B)				
Maternal outcomes	Non-GDM (*n* = 2372)	GDM (*n* = 897)	*P* value	OR^a^ (95% CI)
Preterm birth <34 weeks, *N* (%)	306 (12.9)	127 (14.2)	0.189	0.851 (0.668, 1.083)
Preterm birth <37 weeks, *N* (%)	1306 (55.1)	520 (58.0)	0.534	0.926 (0.728, 1.179)
Hypothyroidism, *N* (%)	240 (10.1)	94 (10.5)	0.969	1.005 (0.777, 1.300)
Preeclampsia, *N* (%)	400 (16.9)	164 (18.3)	0.699	1.041 (0.849, 1.277)
PROM, *N* (%)	520 (21.9)	178 (19.8)	0.406	0.921 (0.759, 1.118)
Placental abruption, *N* (%)	35 (1.5)	12 (1.3)	0.875	0.948 (0.486, 1.848)
Placenta previa, *N* (%)	108 (4.6)	37 (4.1)	0.426	0.854 (0.579, 1.260)
Severe postpartum hemorrhage, *N* (%)	112 (4.7)	44 (4.9)	0.99	1.002 (0.697, 1.441)
Oligohydramnios, *N* (%)	126 (5.3)	43 (4.8)	0.604	0.909 (0.634, 1.304)

(C)			
Maternal characteristics	Non-insulin-treated GDM (*n* = 825)	Insulin-treated GDM (*n* = 72)	*P* value
Age (SD), year	31.31 ± 3.57	32.36 ± 4.04	0.018
< 35, *N* (%)	684 (82.9)	51 (70.8)	0.011
≥ 35, *N* (%)	141 (17.1)	21 (29.2)	
Chorionicity			
MCDA, *N* (%)	106 (12.8)	10 (13.9)	0.801
DCDA, *N* (%)	719 (87.2)	62 (86.1)	
Pre-pregnancy (SD), (kg/m^2^)	22.23 ± 3.06	24.14 ± 2.97	0.013
Underweight, *N* (%)	67 (8.1)	1 (1.4)	0.160^a^
Normal weight, *N* (%)	558 (67.6)	35 (48.6)	<0.001
Overweight, *N* (%)	169 (20.5)	27 (37.5)	0.001
Obesity, *N* (%)	31 (3.8)	9 (12.5)	<0.001
Gestational gain (SD), (kg/m^2^)	6.49 ± 2.39	5.81 ± 2.29	<0.001
Excessive gestational weight gain, *N* (%)	155 (18.8)	11 (15.2)	0.462
Conception by ART, *N* (%)	676 (81.9)	64 (88.9)	0.137
Chronic hypertension, *N* (%)	48 (5.8)	6 (8.3)	0.390

(D)				
Maternal outcomes	Non insulin treated GDM (*n* = 825)	Insulin treated GDM (*n* = 72)	*P* value	OR^a^ (95% CI)
Preterm birth <34 weeks, *N* (%)	113 (13.7)	14 (19.4)	0.053	1.750 (0.994, 3.080)
Preterm birth <37 weeks, *N* (%)	469 (56.8)	51 (70.8)	0.045	2.096 (1.017, 4.321)
Hypothyroidism, *N* (%)	82 (9.9)	12 (16.7)	0.323	1.427 (0.704, 2.893)
Preeclampsia, *N* (%)	154 (18.7)	10 (13.9)	0.227	0.649 (0.321, 1.309)
PROM, *N* (%)	166 (20.1)	12 (16.7)	0.680	0.871 (0.453, 1.677)
Placental abruption, *N* (%)	12 (1.5)	0 (0.0)	-	-
Placenta previa, *N* (%)	32 (3.9)	5 (6.9)	0.323	1.673 (0.603, 4.640)
Severe postpartum hemorrhage, *N* (%)	112 (4.7)	44 (4.9)	0.183	0.255 (0.034, 1.905)
Oligohydramnios, *N* (%)	126 (5.3)	43 (4.8)	0.443	0.564 (0.131, 2.436)

(A) (C) *GDM* gestational diabetes, *MCDA* monochorionic diamniotic twins, *DCDA* dichorionic diamniotic twins, *ART* assisted reproductive techniques

(B) (D) *PROM* premature rupture of membranes

^a^Adjusted odds ratio for maternal age and pre-pregnancy BMI

^a^Fisher’s exact test

We further conducted a subgroup analysis of women with GDM during twin pregnancies based on whether they received insulin therapy or not. We observed that maternal age at delivery ≥35 years (29.2% VS 17.1%, *P* = 0.011), overweight (37.5% VS 20.5%, *P* < 0.001) and obesity (12.5% VS 3.8%, *P* < 0.001) were significantly more prevalent in the women treated with insulin, compared to those managed through diet-and-exercise alone (Table 1C). Additionally, the women requiring insulin treatment were more likely to deliver before 37 weeks of gestation (OR = 2.096, 95% CI = 1.017–4.321, *P* = 0.045) (Table 1D).

In the multiple logistic regression analysis, we identified that maternal age at delivery ≥35 years, overweight, and obesity elevated the risk of developing GDM during twin pregnancies (OR = 1.307, 95% CI = 1.055–1.618, *P* = 0.014; OR = 1.611, 95% CI = 1.315–1.973, *P* < 0.001; OR = 2.951, 95% CI = 1.828–4.762, *P* < 0.001, respectively), and the proportion requiring insulin therapy (OR = 1.881, 95% CI = 1.073–3.295, *P* = 0.027; OR = 2.450, 95% CI = 1.422–4.223, *P* < 0.001; OR = 4.056, 95% CI = 1.728–9.522, *P* < 0.001, respectively) (Table 2). On the other hand, chronic hypertension before pregnancy solely increased the risk of GDM during twin pregnancies (OR = 1.896, 95% CI = 1.290–2.785). Factors, such as chorionicity, conception through ART, hypothyroidism, and pre-eclampsia, did not significantly influence the risk of GDM during twin pregnancies.

**Table 2:** Results of the multiple logistic regression analysis on the risk factors for GDM in women with twin pregnancies

Risk factors	GDM vs Non-GDM		Insulin-treated GDM vs. Non-insulin-treated GDM	
	*P* value	OR (95% CI)	*P* value	OR (95% CI)
Maternal age ≥ 35 years	0.014	1.307 (1.055–1.618)	0.027	1.881 (1.073–3.295)
Underweight	<0.001	0.559 (0.423–0.737)	0.142	0.222 (0.030–1.657)
Overweight	<0.001	1.611 (1.315–1.973)	<0.001	2.450 (1.422–4.223)
Obesity	<0.001	2.951 (1.828–4.762)	<0.001	4.056 (1.728–9.522)
DCDA	0.446	0.898 (0.680–1.185)	0.095	0.492 (0.214–1.131)
Conception by ART	0.113	1.219 (0.955–1.556)	0.162	1.886 (0.775–4.585)
Chronic hypertension	<0.001	1.896 (1.290–2.785)	0.808	0.890 (0.348–2.278)
Hypothyroidism	0.996	1.003 (0.775–1.299)	0.348	1.407 (0.690–2.872)
Preeclampsia	0.501	1.076 (0.876–1.322)	0.192	0.621 (0.303–1.271)

Table 3A presents the neonatal characteristics and outcomes of the study population. We found that compared to those by non-GDM women during twin pregnancies, newborns delivered by women with GDM during twin pregnancies showed a lower rate of birth weight less than 1500 grams (3.1% VS 4.5%, *P* = 0.011), but were more likely to be diagnosed with extrauterine growth restriction (3.2% VS 2.1%, *P* = 0.010). We did not observe significant differences in the mean gestational age and birth weight, sex ratio of newborns, or rates of other adverse neonatal outcomes, including SGA, LGA, birth weight <2500 g, and 1- and 5-min Apgar score.

**Table 3:** (A) Neonatal outcomes of the study population. (B) Neonatal outcomes of women with GDM twin pregnancies (*n* = 897)

(A)				
	Non-GDM (*n* = 4744)	GDM (*n* = 1794)	*P* value	OR (95% CI)
Gestational age (SD), week	35.58 ± 1.94	35.48 ± 1.87	0.051	-
Birthweight (SD), g	2385.63 ± 444.77	2400.79 ± 395.43	0.205	-
Male fetus, *N* (%)	2344 (49.4)	891 (49.7)	0.854	1.010 (0.906, 1.126)
SGA, *N* (%)	610 (12.9)	201 (11.2)	0.070	0.855 (0.722, 1.013)
LGA, *N* (%)	111 (2.3)	49 (2.7)	0.361	1.172 (0.834, 1.648)
Birthweight <1500 g, *N* (%)	212 (4.5)	55 (3.1)	0.011	0.676 (0.500, 0.914)
Birthweight <2500 g, *N* (%)	2607 (55.0)	994 (55.4)	0.742	1.018 (0.913, 1.136)
1-min Apgar score <7, *N* (%)	13 (0.3)	9 (0.5)	0.156	1.835 (0.783, 4.300)
5-min Apgar score <7, *N* (%)	3 (0.1)	1 (0.1)	1.000^a^	0.881 (0.092, 8.479)
Neonatal intensive care unit admission, *N* (%)	127 (2.7)	59 (3.3)	0.184	1.236 (0.903, 1.692)
EUGR, *N* (%)	101 (2.1)	58 (3.2)	0.010	1.536 (1.107, 2.131)
Neonatal hypoglycemia, *N* (%)	57 (1.2)	32 (1.8)	0.070	1.493 (0.965, 2.310)

(B)				
	Non-insulin-treated GDM (*n* = 1650)	Insulin-treated GDM (*n* = 144)	*P* value	OR (95% CI)
Gestational age (SD), week	35.51 ± 1.88	35.13 ± 1.73	0.030	-
Birthweight (SD), g	2396.99 ± 422.56	2358.47 ± 414.62	0.294	-
Male fetus, *N* (%)	816 (49.5)	75 (52.1)	0.545	1.111 (0.790, 1.562)
SGA, *N* (%)	190 (11.5)	7 (4.9)	0.014	0.393 (0.181, 0.852)
LGA, *N* (%)	45 (2.7)	5 (3.5)	0.602	1.283 (0.501, 3.285)
Birthweight <1500 g, *N* (%)	46 (2.8)	3 (2.1)	0.619^a^	0.742 (0.228, 2.416)
Birthweight <2500 g, *N* (%)	912 (55.3)	82 (56.9)	0.699	1.070 (0.759, 1.509)
1-min Apgar score <7, *N* (%)	8 (0.5)	1 (0.7)	1.000^a^	1.435 (0.178, 11.557)
5-min Apgar score <7, *N* (%)	1 (0.1)	0 (0.0)	1.000^a^	-
Neonatal intensive care unit admission, *N* (%)	53 (3.2)	6 (4.2)	0.538	1.310 (0.553, 3.102)
EUGR, *N* (%)	46 (2.8)	12 (8.3)	<0.001	3.170 (1.639, 6.131)
Neonatal hypoglycemia, *N* (%)	31 (1.9)	1 (0.7)	0.483^a^	0.365 (0.049, 2.695)

(A) (B) *GDM* gestational diabetes, *SGA* Small-for-gestational age infants, *LGA* Large-for-gestational age infants, *EUGR* extrauterine growth restriction

^a^Fisher’s exact test

In Table 3B, we presented the neonatal characteristics and outcomes of GDM twin pregnancies stratified by whether the pregnant women received insulin therapy. Newborns of GDM women receiving insulin therapy during twin pregnancies had a significantly lower rate of SGA (4.9% VS 11.5%, *P* = 0.014), but were more likely to have extrauterine growth restriction (OR = 3.170, 95% CI = 1.639–6.131, *P* < 0.001).

## Discussion

It reports that the incidence of women with GDM during twin pregnancies ranges from 3.2% to 25.5%. Advanced maternal age, nulliparity, higher pre-pregnancy BMI, and conception by ART have been identified as risk factors for the development of GDM in women during twin pregnancies [10–12, 14, 16]. In our study, we found that the proportion of maternal age ≥35, overweight and obesity were significantly higher in the women with GDM during twin pregnancies compared to those without, and this proportion further elevated in insulin-treated GDM twin pregnancies. Although we observed a higher incidence of GDM in women with twin pregnancies conceived through ART, the rate of conception by ART was comparable in insulin-treated women. Furthermore, although ART may contribute to GDM during twin pregnancies, and ART twins are more likely to be dichorionic diamniotic, we did not observe a significant difference in the incidence of GDM between twins with different chorionicity.

Some studies have reported an association between GDM and hypertensive complications during twin pregnancies [11, 13–15], but we did not find an increased incidence of pre-eclampsia in women with GDM during twin pregnancies in the present study. The opinions regarding the effect of GDM on hypertensive complications during twin pregnancies are contradictory. Some studies report no significant difference in hypertensive complications between women with and without GDM during twin pregnancies, after adjusting for maternal age, race, IVF treatment, and pre-pregnancy BMI [4, 10]. However, chronic hypertension during twin pregnancies have been shown to increase the risk of GDM [22, 23]. Previous studies have shown that gestational weight gain during twin pregnancies in China ranges from 15 to 21 kg [20, 21]. Excessive gestational weight gain has also been identified as a risk factor for hypertensive complications during pregnancy [24]. Our study found that diet-and-exercise management effectively reduced gestational weight gain in women with GDM during twin pregnancies. This highlights the importance of interventions to control weight gain in all women with twin pregnancies, regardless of the occurrence of GDM.

Previous studies have suggested that GDM does not significantly increase the risk of preterm birth during twin pregnancies, because the preterm birth rate of twin pregnancies has reached a high level, approaching or even exceeding 50% [10, 16, 25]. In our study population, we observed a higher rate of preterm birth (<34 weeks, 14.2% VS 12.9%; <37 weeks, 58.0% VS 55.1%) in GDM twin pregnancies than in non-GDM twin pregnancies, but this rate decreased after adjusting for maternal age and pre-pregnancy BMI (<34 weeks, OR = 0.851; <37 weeks, OR = 0.926), but without significant difference (*P* > 0.05). Furthermore, whether insulin treatment poses a risk on preterm birth during twin pregnancies remains unknown. We observed a significant increase in the preterm birth rate at <37 weeks in insulin-treated GDM twin pregnancies, and this increase remained significant after adjusting for maternal age and pre-pregnancy BMI (OR = 2.096, 95% C= (1.017,4.321). In our study cohort, besides preterm birth, we did not find significant differences in other maternal outcomes between GDM and non-GDM twin pregnancies.

Our study found that newborns from women with GDM during twin pregnancies had similar neonatal outcomes as those from women without, which is consistent with previous studies [5, 16]. However, we observed that newborns from insulin-treated women for GDM during twin pregnancies, though having a smaller average gestational age and birth weight, still presented a lower incidence of SGA, suggesting a possible protective effect of higher blood glucose levels against SGA [18, 26, 27]. Conversely, we also found that although the incidence of SGA in newborns from insulin-treated women was lower, the incidence of EUGR was significantly higher. EUGR may impose long-term serious impact on the development of nervous and many other systems in premature newborns [28]. Previous studies have shown that gestational age <34 weeks, birthweight <1500 g, and SGA are independent risk factors for the development of EUGR in newborns [29–31]. However, our study did not find significant differences in these factors. The hyperglycemia-hyperinsulin theory suggests that high blood glucose levels in pregnant women with GDM can induce fetal hyperglycemic, thus stimulating fetal pancreatic β-cell enlargement and insulin hypersecretion. Insulin, a main driver in fetal growth, protein and fat synthesis, can inhibit lipolysis to boost excessive fetal growth and development, thereafter reducing the incidence of SGA during the second and third trimesters of pregnancy [32, 33].

Prior research has demonstrated that infants of diabetic mothers (IDMs) exhibit a 50% increase in fat mass, compared to infants born to women without GDM. Consequently, the fat-free weight is lower in IDMs with the same birth weight [34]. In premature newborns, the amount of nutrition required for catch-up growth therapy is calculated based on gestational age and fat-free weight. Insufficient calorie supply may fail an anticipated growth and lead to extrauterine growth restriction. In addition, hyperinsulinemia can hinder fetal organ maturation, especially when combined with placental microvascular abnormalities resulting from hyperglycemia. Hickman et al. have demonstrated that maternal glycemic control is necessary for achieving optimal outcomes in insulin therapy for pregnant women with GDM [26]. However, maternal glycemic control with insulin does not prevent GDM-associated fetoplacental vascular and metabolic alterations. Few reports have mentioned that insulin-treated pregnant women may face a low risk of fetoplacental endothelial dysfunction [27]. Although insulin-treated pregnant women and their newborns may exhibit normal blood glucose levels at delivery, vascular dysfunction in the placenta has been observed [35, 36]. It is now understood that after birth, the hyperglycemic state disappears, and insulin secreted by the enlarged islet β-cells continue to lower blood glucose levels in the neonatal period, resulting in EUGR [37]. In the present study, we observed that insulin-treated women with GDM during twin pregnancies had significantly lower gestational weight gain, compared to those receiving no insulin treatment. However, it should keep in mind that the occurrence of EUGR may be associated with inadequate nutritional intake of women with insulin-treated GDM.

Interestingly, insulin-treated GDM may increase the risk of long-term health issues in twin newborns, due to the higher incidence of premature birth and EUGR. Recent data demonstrate that fetal overgrowth may appear prior to the traditional diagnosis of GDM, and the durable adverse impact of maternal hyperglycemia on child and adolescent metabolism [38–40].

Early identification of GDM is crucial, because if intervention is postponed to the later stage of pregnancy, the fetus may have already been harmed, but not yet diagnosed. The Chinese Medical Association guidelines recommend that all pregnant women should undergo a fasting blood glucose test during their first antenatal examination (10–14 gestational weeks), and early dietary guidance should be provided in the presence of impaired fasting glucose (IFG) [1]. Early identification and intervention of GDM can help prevent adverse outcomes and benefit the long-term health of twin newborns.

### Strengths and limitations

Our study has several strengths, such as a large sample size from a single institution and uniformed diagnostic methods for GDM over the study period. We included a significant number of twins conceived by ART, and their good compliance allowed for standardized implementation of diet-and-exercise management. We excluded pre-existing diabetes and multiparas to eliminate confounding factors in pregnancy outcomes. Additionally, we divided pregnant women with GDM into two cohorts according to their insulin treatment status, which had been rarely seen in previous studies. However, our study also has some limitations. First, due to its retrospective design, some factors essential for the development of GDM, such as OGTT values, blood glucose control level, and insulin dosage, were not analyzed. Second, we excluded samples with fetal structural abnormalities, despite reports showing that GDM increases the risk of fetal malformations [11]. Moreover, we did not compare the rate of cesarean section between women with and without GDM during twin pregnancies, as cesarean section is always preferred for women with twin pregnancies during the study period according to Chinese guidelines. Finally, we did not track the long-term outcomes of the mothers and their infants.

## Conclusions

Our study identified significant risk factors associated with GDM in women during twin pregnancies, including advanced maternal age and pre-pregnancy overweight. Chronic hypertension may also increase the risk of GDM. We found that women with GDM during twin pregnancies had pregnancy and neonatal outcomes similar to those without. However, women with GDM during twin pregnancies receiving insulin therapy were more prone to preterm birth, and their newborns had a higher incidence of EUGR. Our findings provide novel insights into the risk and outcomes of GDM during twin pregnancies and may guide the development of effective interventions.

### Supplementary information

Supplementary Table 1

## Data Availability

Data supporting the results of this study can be obtained on request to the author Yuan Yuan (cq_double_yy@163.com).
