# Supplementary material for: Maternal and neonatal outcomes of twin pregnancies complicated by gestational diabetes mellitus
Source: Endocrine. 2023 Nov 10;84(2):388–98. doi: 10.1007/s12020-023-03588-0 (PMC11076322; doi:10.1007/s12020-023-03588-0)
Supplement: Supplementary file 1 — Supplementary Table 1 [file 12020_2023_3588_MOESM1_ESM.docx]

Supplementary Table 1 Gestational weight gain in different pre-pregnancy BMI groups

| Pre-pregnancy BMI | Non-GDM (n=2372) | GDM (n=897) | *P value* | Insulin-treated GDM (n=72) | *P value* |
| --- | --- | --- | --- | --- | --- |
| Underweight | 18.25±5.92 | 16.69±5.02 | 0.043 | 19 | 0.636 |
| Normal weight | 17.56±5.14 | 16.53±5.45 | <0.001 | 14.75±5.78 | 0.382 |
| Overweight | 16.86±5.81 | 15.71±7.82 | 0.055 | 13.98±5.73 | 0.267 |
| obesity | 15.89±6.45 | 13.47±6.09 | 0.104 | 11.06±7.01 | 0.290 |
